# X-ray reflectivity measurement of interdiffusion in metallic multilayers during rapid heating

**DOI:** 10.1107/S1600577517008013

**Published:** 2017-06-15

**Authors:** J. P. Liu, J. Kirchhoff, L. Zhou, M. Zhao, M. D. Grapes, D. S. Dale, M. D. Tate, H. T. Philipp, S. M. Gruner, T. P. Weihs, T. C. Hufnagel

**Affiliations:** a Department of Materials Science and Engineering, Johns Hopkins University, Baltimore, MD 21218, USA; b School of Materials Science and Engineering, Beijing Institute of Technology, Beijing 100081, People’s Republic of China; c Cornell High Energy Synchrotron Source (CHESS), Cornell University, Ithaca, NY 14853, USA; d Department of Physics, Cornell University, Ithaca, NY 14853, USA; e Kavli Institute at Cornell for Nanoscale Science, Cornell University, Ithaca, NY 14853, USA

**Keywords:** X-ray reflectivity, multilayer, interdiffusion

## Abstract

A technique for measuring interdiffusion in multilayer materials during rapid heating using X-ray reflectivity is described. In this technique the sample is bent to achieve a range of incident angles simultaneously, and the scattered intensity is recorded on a fast high-dynamic-range mixed-mode pixel array detector. Heating of the multilayer is achieved by electrical resistive heating of the silicon substrate, monitored by an infrared pyrometer. As an example, reflectivity data from Al/Ni heated at rates up to 200 K s^−1^ are presented. At short times the interdiffusion coefficient can be determined from the rate of decay of the reflectivity peaks, and it is shown that the activation energy for interdiffusion is consistent with a grain boundary diffusion mechanism. At longer times the simple analysis no longer applies because the evolution of the reflectivity pattern is complicated by other processes, such as nucleation and growth of intermetallic phases.

## Abstract 2

A method for *in situ* X-ray reflectivity measurements on the millisecond time scale is described, and its use for measuring interdiffusion in metallic multilayers is illustrated.

## 1. Introduction   

Solid-state interdiffusion is of profound importance in nanostructured materials, where the diffusion distances are short and diffusion times can be small. In semiconductor electronics, for example, the possibility of device failure resulting from interdiffusion has spurred extensive research into materials that can act as diffusion barriers between device components (Nicolet, 1997 ▸). Because common analysis techniques either require destructive depth profiling or are rather slow, most studies of interdiffusion are performed *ex situ*, often after isothermal annealing. Such studies usually assume that the transients associated with heating and cooling the specimen can be neglected.

In some situations, however, the transients themselves are of interest, for example in rapid thermal annealing. In other situations, interfacial reactions alter the structure of the material in ways that make the interpretation of interdiffusion measurements after the fact difficult or impossible. It is therefore desirable to develop experimental techniques that allow measurements *in situ*, while interdiffusion is occurring.

One such technique is X-ray reflectivity (low-angle diffraction) performed on multilayer materials. In the low-angle region the intensities of X-ray scattering peaks are related to the composition modulations through the thickness of the multilayer. By monitoring the change in the intensities of these peaks we can measure interdiffusion. In fact, X-ray reflectivity is among the most sensitive probes of interdiffusion, capable of measuring interdiffusion coefficients as low as 10^−27^ m^2^ s^−1^ (Greer, 1997 ▸).

In a conventional X-ray reflectivity measurement the scattered intensity is recorded by step-scanning through a range of angles. This makes *in situ* observations impractical except for slow processes. To overcome this limitation, several techniques have been developed for recording complete reflectivity patterns simultaneously. For example, using a curved specimen one can record the scattering over a range of angles simultaneously using a position-sensitive X-ray detector (Naudon *et al.*, 1989 ▸; Niggemeier *et al.*, 1997 ▸; Stoev & Sakurai, 2013 ▸). Another approach is to use an X-ray beam with a range of wavelengths, either by dispersing the X-ray beam into a range of angles (Matsushita *et al.*, 2008 ▸) (again using a position-sensitive detector) or, at a single angle, recording scattering with an energy-sensitive detector (Neissendorfer *et al.*, 1999 ▸; Raghavendra Reddy *et al.*, 2009 ▸).

In this paper we show how to use the curved-sample approach to perform time-resolved *in situ* X-ray reflectivity characterization of the initial stages of interdiffusion during continuous heating of metallic multilayers. We have tested our technique at heating rates up to 200 K s^−1^, but in principle it can be applied at much higher rates, limited by the intensity of the X-ray source and the capabilities of the X-ray detector. During the initial stages of heating we can determine the interdiffusion coefficient 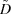 by a simple analysis of the rate of decay of the peaks in the reflectivity pattern, which are related to the composition modulation of the multilayer. At longer times this simple analysis becomes unreliable because the reflectivity pattern is affected by other processes, such as nucleation and growth of intermetallic phases.

## 2. Experimental   

**Figure 1:**
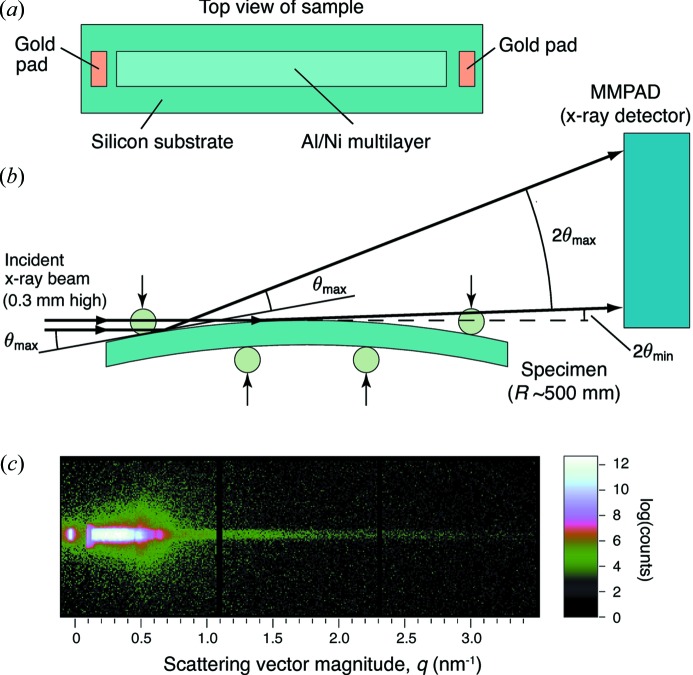
(*a*) The samples had lithographically patterned regions of Al/Ni multilayer (4 cm long by 1 cm wide) along with gold pads (0.5 cm by 1 cm) to act as electrical contacts. (*b*) The samples were dynamically bent in a four-point loading apparatus, with the curvature of the specimen measured by a laser wafer-curvature technique and temperature monitored by an optical pyrometer. (*c*) Example raw data from a 3 × 1 section of chips on the MMPAD array.

The samples for this study were multilayer foils produced by DC magnetron sputtering alternating layers of alluminum alloy 1100 with layers of nickel–7 wt% vanadium. The ratio of the Al layer thickness to the Ni–V layer thickness was 3:2, which yields an atomic ratio of Al:Ni–V of 1:1. The bilayer period of the layers (*i.e.* the sum of one Al and one Ni–V layer thickness) was 20–30 nm. Because X-ray reflectivity is strongly sensitive to the roughness of the layers and the roughness increases with the number of layers deposited, we restricted the thickness of our samples to three to six bilayer periods (Al/Ni layer pairs). The multilayers were deposited onto 500 µm-thick polished Si wafers, onto which we also deposited 300 nm-thick gold pads by thermal evaporation (Fig. 1*a* ▸). (A 20 nm-thick layer of tungsten was deposited first to promote adhesion of the gold to the silicon substrate.) The gold pads provided electrical contacts to permit resistive heating of the doped Si substrate, which in turn heated the multilayer. The power source for these experiments was a series array of 9 V or 12 V batteries, switched with a solid-state relay to allow control of the duration of the current pulse. We monitored the temperature of the multilayer during heating with a single-wavelength infrared pyrometer (Kleiber KGA 740-LO), sampling at 50 Hz. The lowest temperature that can be measured with this pyrometer is 475 K, so we were unable to use it to track the very earliest stages of heating.

Angle-dispersive X-ray reflectivity uses curved specimens so that the angle of incidence of X-rays on the surface varies with position, as shown in Fig. 1(*b*) ▸. Although multilayers can be deposited on curved substrates, we elected instead to use flat substrates which we then bent in a specially designed loading fixture. Pragmatically, flat substrates are cheaper than precisely polished curved substrates, and it is easier to deposit uniform multilayers on them. This approach also allows flexibility in choosing the radius of curvature (and thus the range of angles over which the reflectivity is measured). Our four-point bending apparatus is illustrated in Fig. 1(*b*) ▸. The two lower loading rods are fixed in position, and bending is achieved by using stepper motors to displace the two upper rods. Gaps in the upper rods (not shown) provide a clear path for the X-rays, and the rods (which are made of steel) are electrically isolated from the substrate by kapton tape. Prior to each reflectivity measurement we measured the curvature of the specimen by means of a parallel-beam curvature setup similar to that described by Floro *et al.* (1996 ▸).

The experiments described here were performed at station A2 of the Cornell High Energy Synchrotron Source (CHESS) using a Si(111) double-crystal monochromator to select 12.0 keV X-rays, with a flux of approximately 5 × 10^10^ photons s^−1^ mm^−2^. The beam height (0.3 mm) yielded a range of incident angles from zero to approximately 1.3°, depending on the radius of curvature of the specimen (typically about 500 mm). The X-ray detector was a mixed-mode pixel array detector (MMPAD), which is capable of framing continuously at high rates (up to 1 kHz) and has a large dynamic range (>4 × 10^7^ photons pixel^−1^) (Tate *et al.*, 2013 ▸). The dynamic range is useful because it allows both the low- and high-order scattering peaks, which can differ in intensity by many orders of magnitude, to be measured simultaneously. The detector used here employed a 3 × 2 array of MMPAD chips (with each chip having a square 128 × 128 array of 150 µm pixels) although only a single row of three chips was used for these measurements (Fig. 1*c* ▸). With this detector placed 1013 mm from the sample we recorded scattering over a range of scattering vectors (*q* = 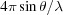, where θ is one-half of the scattering angle and λ is the X-ray wavelength) of about 3 nm^−1^. The width of the X-ray beam was 1 mm, and the one-dimensional reflectivity patterns shown below were obtained by simply summing the output from the detector across the width of the beam at each row of pixels corresponding to a given value of *q*.

**Figure 2:**
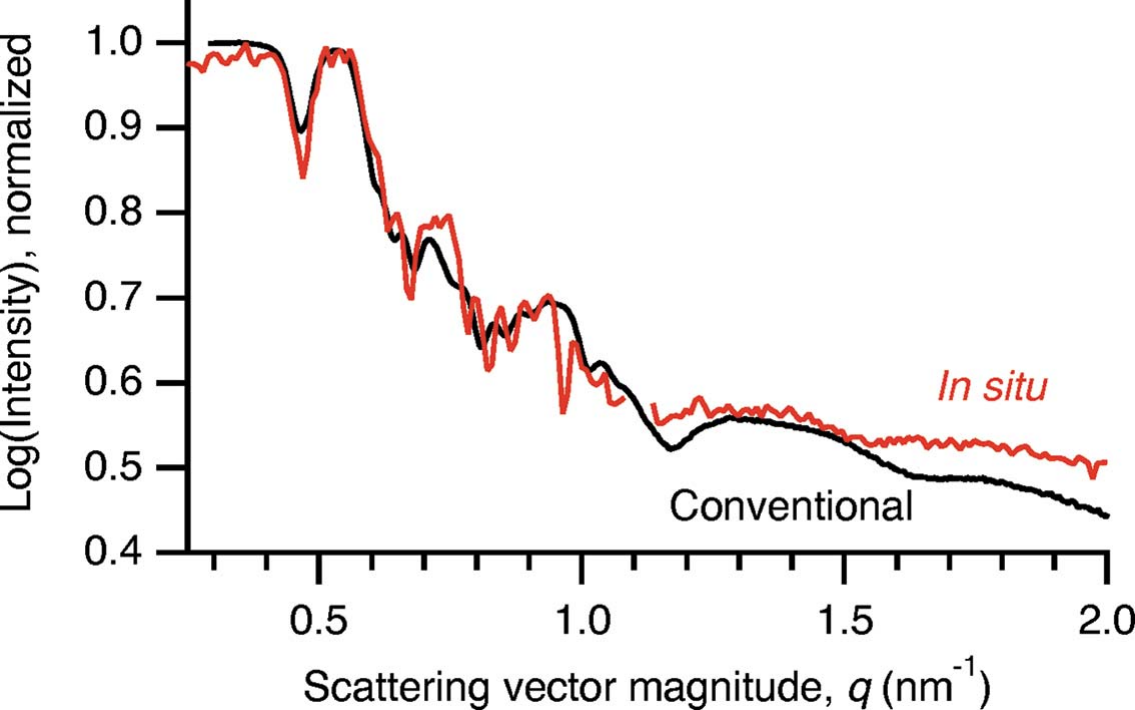
Comparison of reflectivity profiles measured with the *in situ* apparatus and a conventional laboratory diffractometer. The intensities have been normalized to match in the total external reflection region at small *q*. Here, and in the other figures, log refers to base-10 logarithm while ln refers to natural (base *e*) logarithm

Fig. 2 ▸ shows X-ray reflectivity data from an Al/Ni multilayer sample with a nominal bilayer period of 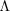 = 20 nm, recorded two ways: using the apparatus described above, and on a conventional parallel-beam diffractometer (Philips MRD) using Cu *K*α radiation with the sample nominally flat. Although the basic features of the laboratory reflectivity data are reproduced in the *in situ* synchrotron experiment, the agreement is not perfect. There are several reasons for this. First, the *in situ* technique records some non-specular scattering (θ_incident_ ≠ θ_exit_) in addition to the desired specular scattering (θ_incident_ = θ_exit_). Second, there is variation in the incident intensity in the *in situ* case due to the intensity profile along the height of the synchrotron beam. Third, no attempt has been made to correct for geometrical aberrations such as anticlastic bending of the substrate. Finally, the energy bandpass and the angular divergence of the X-ray beam are different between the two cases.

Despite these differences the two reflectivity profiles are in reasonably good agreement. Both profiles show an intensity plateau below about *q* = 0.4 nm^−1^ which is due to total external reflection of X-rays from the multilayer. Above this, both profiles show a series of low-angle scattering peaks from the multilayer structure.

## 3. Results and discusson   

**Figure 3:**
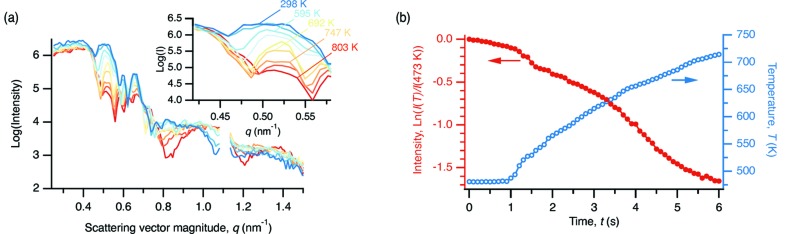
(*a*) Evolution of the X-ray reflectivity of an Al/Ni multilayer with nominal bilayer period 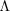 = 25 nm during heating at 40 K s^−1^. Each exposure was 100 ms. The inset shows the first low-angle scattering peak in more detail. (*b*) Evolution of peak intensity, plotted as ln[*I*(*T*)/*I*(473 K)], with time for determination of 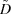.

Fig. 3(*a*) ▸ shows reflectivity data from an Al/Ni multilayer recorded *in situ* during heating at 40 K s^−1^. The intensity of the first peak (shown in the inset) decreases with increasing temperature. The amplitudes of the peaks in the low-angle region are proportional to the square of the amplitude of the composition modulation (Cook, 1969 ▸; Paulson & Hilliard, 1977 ▸). If no phase transformation occurs (a point to which we return below) as interdiffusion proceeds, the composition of the multilayer becomes more uniform and the intensity of the low-angle peaks decreases.

For the more common case of interdiffusion studied at constant temperature, the intensity of a low-angle scattering peak 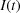 at time *t* is related to that at initial time 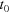 by
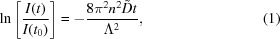
where *n* is the order of the reflection and 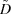 is the interdiffusion coefficient (Wang *et al.*, 1999 ▸). The bulk interdiffusion coefficient 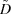 can therefore be determined from the slope of a plot of 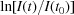 against *t*.

There are several issues with applying equation (1) to the present case. First, our experiments were conducted at constant heating rate. We make the assumption that because our heating rates are high and 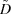 increases exponentially with temperature, the amount of interdiffusion that occurs at temperature *T* over a given interval 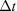 is large compared with the amount of interdiffusion that occurred in heating up to that temperature. This assumption is easily checked by comparing the integral of the diffusion equation for constant heating rate with that for isothermal interdiffusion (given the activation energy for interdiffusion) (Khawam & Flanagan, 2006 ▸). This assumption allows us to recast equation (1) in terms of temperature and write
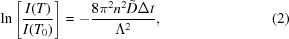
where 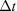 is the interval between measurements (100 ms for the experiments described here). Fig. 3(*b*) ▸ shows a plot of 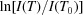 against *t*, for 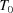 = 473 K.

Another potential complication in the application of equation (1) for measuring interdiffusion in multilayers is that it does not apply to situations in which the concentration gradients are very steep. As discussed by Greer & Spaepen (1985 ▸), however, this effect is small when the bilayer period 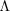 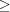 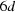, where *d* is the atomic spacing parallel to the diffusion direction. The interplanar spacings of Al and Ni are around 2.0–2.3 Å [for the Ni(111) and Al(111) planes, respectively], much smaller than the bilayer periods used here (20–30 nm). Furthermore, the substantial intermixing that occurs during sputter deposition of Al/Ni multilayers (Gavens *et al.*, 2000 ▸) acts to reduce the concentration gradient. Therefore, equation (1) can be applied without an explicit correction for the effect of the concentration gradient.

**Figure 4:**
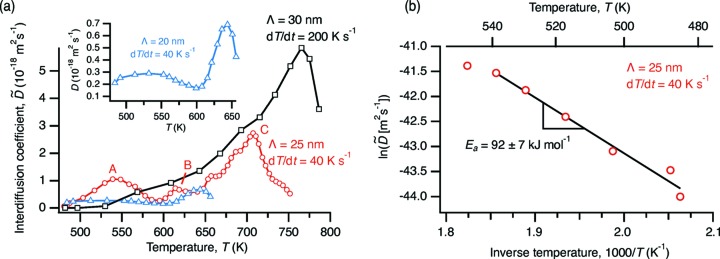
(*a*) Interdiffusion coefficient 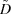 for several combinations of bilayer period and heating rate. Labels *A*, *B* and *C* identify the peaks in 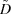 for the multilayer with 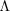 = 25 nm discussed in the main text and referenced in Fig. 5 ▸. The inset shows the data for 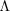 = 20 nm in more detail. (*b*) Arrhenius plot for determination of activation energy *E*_a_ for the 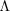 = 25 nm sample from part (*a*). The fit is to the five data points from the lowest temperature range (485 K to 530 K).

Fig. 4(*a*) ▸ shows the interdiffusion coefficient 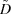 as a function of temperature, determined from the decay of the low-angle scattering peaks using equation (2), for several combinations of bilayer period and heating rates. We begin by focusing our attention on the low-temperature end of Fig. 4(*a*) ▸. If the evolution of the composition profile is dominated by a single thermally activated diffusion mechanism, then 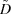 should increase exponentially with temperature. To check this, Fig. 4(*b*) ▸ shows an Arrhenius plot of 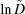 *versus* 1/*T* for the 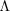 = 25 nm multilayer heated at 40 K s^−1^. We see that at the lowest temperatures the behavior is indeed linear, with an apparent activation energy for interdiffusion of *E*_a_ = 92 ± 7 kJ mol^−1^. Data for the 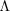 = 30 nm multilayer heated at 200 K s^−1^ yield a similar value, *E*_a_ = 80 ± 19 kJ mol^−1^. We were not able to extract a reliable activation energy for the smallest bilayer period (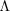 = 20 nm) due to substantial interdiffusion that occurred during heating up to the lowest temperature at which we could make reliable pyrometer measurements (475 K).

Du and co-workers (Du *et al.*, 2003 ▸) performed a critical assessment of bulk interdiffusion coefficients in a variety of systems and reported an activation energy of *E*_a_ = 144.6 kJ mol^−1^ for diffusion of Ni in face-centered-cubic Al, based on indirect observations by Erdélyi and co-workers (Erdélyi *et al.*, 1978 ▸). If we assume that interdiffusion is dominated by diffusion of nickel [because nickel is known to be a fast diffuser in aluminium (Edelstein *et al.*, 1994 ▸)], as a rough approximation we may also take this value of activation energy as representative of interdiffusion. For Ni-rich alloys, Watanabe and co-workers reported higher activation energies for interdiffusion, *E*_a_ = 214–277 kJ mol^−1^ (Watanabe *et al.*, 1994 ▸). Taken together, this prior work, though limited, suggests an activation energy for interdiffusion of roughly *E*_a_ = 200 ± 50 kJ mol^−1^.

However, those earlier measurements were from higher temperatures [742–924 K (Erdélyi *et al.*, 1978 ▸) and 1050–1400 K (Watanabe *et al.*, 1994 ▸)] than those we used for our determination of *E*_a_ (Fig. 4*b* ▸). It is reasonable to suspect that grain boundary diffusion may dominate at lower temperatures, particularly for our multilayers where the grain size is of the order of a few nanometers. If we assume that this is the case, and that the activation energy for grain boundary diffusion is about half that for lattice diffusion [based on an average value for face-centered-cubic metals (Brown (1980 ▸)], we arrive at a value of around 100 ± 25 kJ mol^−1^. This is consistent with recent measurements (at temperatures similar to ours) by Grieseler and co-workers who reported *E*_a_ = 120 kJ mol^−1^ and also assumed a grain boundary diffusion mechanism (Grieseler *et al.*, 2014 ▸). Another point of comparison is an activation energy for solid-state interdiffusion of 77 ± 1 kJ mol^−1^ calculated by Fritz and co-workers based on the ignition threshold for self-propagating reactions in Al/Ni–V multilayers very similar to those considered here (Fritz *et al.*, 2013 ▸). All of these numbers are reasonably consistent with our measured values of ∼90 kJ mol^−1^.

**Figure 5:**
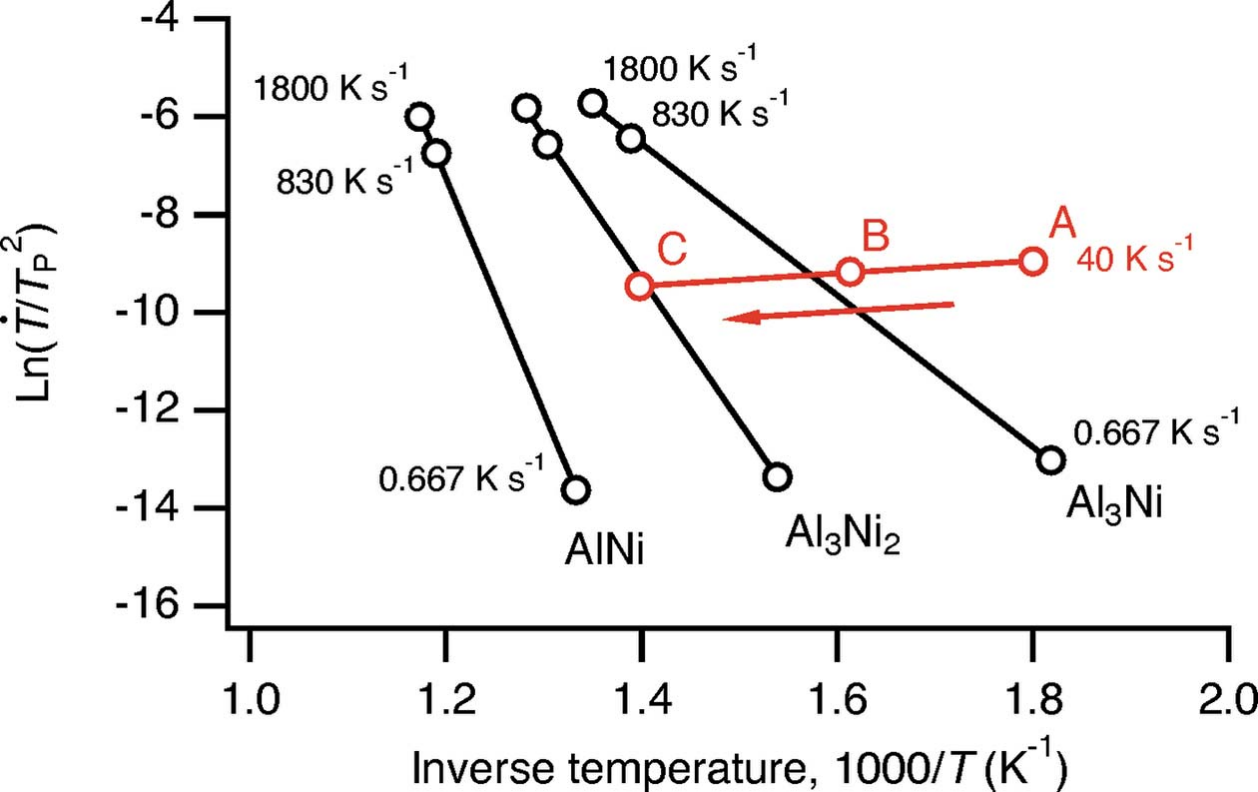
Kissinger plots for the formation of the intermetallic phases Al_3_Ni, Al_3_Ni_2_ and AlNi, based on DSC data from Grapes (2016) ▸. Points *A*, *B* and *C* refer to the temperatures at which peaks in 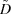 occur for the 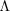 = 25 nm sample (Fig. 4*a* ▸). Joining them is a line indicating a heating rate of 40 K s^−1^; the arrow indicates the direction of increasing temperature along this line. At this heating rate, peak *B* occurs at a temperature close to that which would be expected for formation of Al_3_Ni and peak *C* near the temperature expected for Al_3_Ni_2_.

After this initial stage of interdiffusion during which 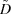 increases exponentially with temperature, more complex behavior is observed at higher temperatures (Fig. 4*a* ▸). This complexity presumably results from other processes that also affect the composition profile of the multilayer. Al/Ni multilayers undergo a series of intermetallic formation reactions with increasing temperature, the details of which depend on the overall composition, bilayer period and heating rate (Knepper *et al.*, 2009 ▸; Grapes *et al.*, 2014 ▸). Activation energies for phase transformations occurring at constant heating rate are commonly determined using the Kissinger equation,
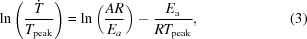
where 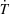 is the heating rate, *T*_peak_ is the temperature at the peak maximum in a DSC scan, *A* is a pre-exponential constant, *R* is the gas constant and *E*_a_ is the activation energy. Data drawn from Grapes (2016 ▸) for the formation of Al_3_Ni, Al_3_Ni_2_ and AlNi at various heating rates are shown in Fig. 5 ▸. Also indicated on this plot are the temperatures of the three peaks in 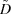 labeled *A*, *B* and *C* in Fig. 4(*a*) ▸ for the 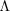 = 25 nm sample heated at 40 K s^−1^. Peaks *B* and *C* appear at temperatures close to those expected for the formation of Al_3_Ni and Al_3_Ni_2_, respectively. The decrease in apparent 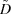 above these temperatures may or may not be real. The intermetallic phases act as diffusion barriers and reduce 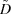, although we note that these phases also have grain boundaries which would reduce their effectiveness as diffusion barriers. Alternatively, the formation of the intermetallic phases may affect the reflectivity pattern in ways that only make it appear that 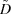 is decreasing.

Peak *A*, on the other hand, occurs at a lower temperature than would be expected for formation of any intermetallic phase at this heating rate. In our view, this behavior most likely results from the composition dependence of 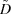. In particular, if grain boundary diffusion dominates at low temperatures then it may be that the Al grain boundaries quickly become saturated with Ni, which would slow down subsequent diffusion.

## 4. Conclusions   

We have demonstrated an X-ray reflectivity technique for measuring solid-state interdiffusion in multilayer materials during rapid heating. Here we have demonstrated the technique at rates of up to 200 K s^−1^ but it could readily be extended to higher rates. We have, for example, collected reflectivity patterns with reasonably good signal-to-noise ratios in as little as 2 ms, implying the ability to collect data at heating rates as fast as 10^4^ K s^−1^. More detailed studies, for example of interlayer roughness, may also be possible from a more complete consideration of the two-dimensional reflectivity profiles (Fig. 1 ▸). This would require careful corrections for the intensity profile of the incident beam and geometrical aberrations due to the curved sample (Stoev & Sakurai, 2013 ▸), along with modeling of the specular and diffusion scattering from the specimens.
